# A study on the sustainability assessment of China’s basic medical insurance fund under the background of population aging–evidence from Shanghai

**DOI:** 10.3389/fpubh.2023.1170782

**Published:** 2023-06-02

**Authors:** Zhi-Qing Yu, Li-Peng Chen, Jun-Qiao Qu, Wan-Zong Wu, Yi Zeng

**Affiliations:** ^1^School of Public Administration, Zhongnan University of Economics and Law, Wuhan, China; ^2^Business School, Yangzhou University, Yangzhou, China

**Keywords:** medical insurance for employees, fund sustainability, population aging, China, actuarial model

## Abstract

**Objective:**

As China’s population aging process accelerates, the expenditure of China’s basic medical insurance fund for employees may increase significantly, which may threaten the sustainability of China’s basic medical insurance fund for employees. This paper aims to forecast the future development of China’s basic medical insurance fund for employees in the context of the increasingly severe aging of the population.

**Methods:**

This paper taking an empirical study from Shanghai as an example, constructs an actuarial model to analyze the impact of changes in the growth rate of *per capita* medical expenses due to non-demographic factors and in the population structure on the sustainability of the basic medical insurance fund for employees.

**Results:**

Shanghai basic medical insurance fund for employees can achieve the goal of sustainable operation in 2021-2035, with a cumulative balance of 402.150–817.751 billion yuan in 2035. The lower the growth rate of *per capita* medical expenses brought about by non-demographic factors, the better the sustainable operation of the fund.

**Conclusion:**

Shanghai basic medical insurance fund for employees can operate sustainably in the next 15 years, which can further reduce the contribution burden of enterprises, which lays the foundation for improving the basic medical insurance treatment for employees.

## 1. Introduction

According to the Sixth Census of China in 2010, the proportion of people aged 65 and above in China reached 8.87%, and this proportion increased to 13.5% in 2020, further aggravating the aging of China’s population. The rapid paces of population aging will have an impact on China’s basic medical insurance fund for urban employees (hereinafter referred to as “basic medical insurance fund for employees”), as the older adults spend more on medical services than the young (1). Based on data published by China’s Ministry of Human Resources and Social Security and National Healthcare Security Administration, the total expenditure of China’s basic medical insurance fund for employees increased from 327.160 billion yuan in 2010 to 1283.399 billion yuan in 2020, 3.92 times that of 2010 (Table 1). This is due to the fact that on the one hand, the increase in the proportion of the older adults population leads to an increase in medical expenses and a relative expansion of the medical insurance fund expenditure. On the other hand, given that retired employees do not contribute to medical insurance, the proportion of young people decreases and the number of insured contributors decreases as a result, which leads to a relative reduction in the fund income, increasing the pressure on the fund operations and putting its sustainability to the test.

**Table 1:** Income and expenditure of the basic medical insurance fund for employees from 2010 to 2020.

Year	China			Shanghai		
	Income	Expenditure	Balance	Income	Expenditure	Balance
2010	395.540	327.160	68.380	32.977	30.007	2.970
2011	494.500	401.800	92.700	41.970	32.649	9.321
2012	606.200	486.800	119.400	53.967	36.333	17.634
2013	706.160	582.990	123.170	61.564	40.919	20.645
2014	803.790	669.660	134.130	66.473	46.807	19.666
2015	908.350	753.150	155.200	75.015	51.894	23.121
2016	1027.370	828.670	198.700	86.760	57.192	29.568
2017	1227.830	946.690	281.140	135.802	68.138	67.664
2018	1325.928	1050.492	275.436	113.394	82.414	30.980
2019	1584.500	1266.300	318.200	126.905	83.299	43.606
2020	1562.461	1283.399	279.062	123.549	97.230	26.319

Data source: Ministry of Human Resources and Social Security of the People’s Republic of China, National Healthcare Security Administration, Shanghai Healthcare Security Bureau. Unit: billion yuan.

This paper assesses the sustainability of China’s basic medical insurance fund in the context of population aging using an empirical study from Shanghai as an example. There are two reasons for using the Shanghai sample, one is at present, China’s basic medical insurance schemes pool at provincial level, but not yet at the national level, so the empirical study is conducted on a provincial basis. Another reason is that Shanghai is one of the first cities to enter the aging society in China, and its population aging is relatively serious (2). According to National Bureau of Statistics of the People’s Republic of China (3, 4), Shanghai’s population aged 65 and above was 11.53% in 2000, and in 2020 this proportion reached 16.28%, exceeding the national average of 13.5%, and nearly 5 percentage points higher than that in 2000, which illustrates that the degree of population aging in Shanghai is rapidly deepening. In accordance with international practice,^1^ Shanghai is currently at a moderate aging degree, and the deepening trend is outstanding, mainly because of its low fertility rate, combined with the conversion of the baby boomers born in Shanghai in the 1950s into the current older adults population, which will have an impact on Shanghai basic medical insurance fund for employees. Table 1 reflects the results of the income and expenditure of Shanghai basic medical insurance fund for employees from 2010 to 2020, which shows that the expenditure of the Shanghai basic medical insurance fund for employees increases year by year.

It can be seen that China’s population aging is deepening and the payment pressure on China’s basic medical insurance fund is gradually increasing, which has certain impact on the sustainability of the fund. If China’s basic medical insurance fund is not sustainable, fiscal pressure will increase, and the government will try to expand tax revenue, making the tax burden heavier, and possibly even leading to government bankruptcy (5, 6). Government bankruptcy has occurred in some countries and regions, but it has not yet occurred in China (7). Therefore, the sustainable development of the basic medical insurance fund is extremely important for the development of the national economy, and if the income and expenditure balance of the medical insurance fund is not stabilized, it will certainly cause damage to the national economy. It is of great practical significance to forecast the future development of China’s basic medical insurance fund in the context of the increasingly severe aging of the population.

Our research tries to answer two questions: whether the changes of population age structure brought about by aging can achieve sustainable operation of the fund? What will be the impact of non-demographic increases in medical expenses on fund sustainability? Therefore, we use actuarial model to predict the impact of medical expense changes caused by demographic structure changes and non-demographic factors on the operation of China’s basic medical insurance fund in the future. Using the data of Shanghai, we set three population projection scenarios, i.e., low, medium and high, to simulate the change of population structure. Also we set three scenarios, i.e., the growth rate of *per capita* medical expenses caused by non-demographic factors is 2% faster, 1% faster than the actual contribution base growth rate and equal, to simulate the change of *per capita* medical expenses growth rate caused by non-demographic factors. We expect that the conclusions of this research can promote the sustainable development of China’s basic medical insurance fund, and provide empirical reference for other countries and regions in the world with the same level of population aging as China.

## 2. Literature review

As regards the implications of aging upon health care expenditure(HCE), scholars have conducted extensive research and most of them agree that aging brings about an increase in HCE. Scholars differ on whether aging is a key driver of HCE. Some scholars believe that aging is one of the key drivers of HCE. Boz and Ozsari (8) found a unidirectional causal relationship between population aging and HCE using data from Turkey from 1975 to 2016, with fertility leading to population aging. Cho and Kim (9) expected the population of South Korea to continue aging in the future, with HCE gradually increasing as the proportion of the population aged over 65 years increases. Shakoor et al. (10) analyzed the impact of population aging on HCE in Pakistan using time series data for the period 1995 to 2014 and found that population aging and increasing life expectancy led to a significant increase in HCE. Mohapatra et al. (11) identified a significant positive effect of population aging on *per capita* HCE in India over the period 1981–2018.

However, some scholars argue that demographic factors are not the key driver of rising HCE. Bech et al. (12) pointed out that life expectancy was a more important driver of HCE, leading to an exponential increase in HCE, and indicated that aging has a positive short-term effect on HCE. The study of Wong et al. (13) for Netherlands and Howdon and Rice (14) for America concluded that there was a clear effect of proximity to death on HCE and the impact of age on HCE is modest when compared to proximity to death. Harris and Sharma (15) found that Aging had a direct impact on the growth of HCE, but this impact was likely to be dwarfed by other demand and supply factors. HCE projections for OECD countries by Lorenzoni et al. (16) indicated that income was the main driver of HCE growth, with demographic effects accounting for about a quarter of the growth. Costa-Font and Levaggi (17) identified that aging alone was not a powerful HCE driver, but the combined effect of costly innovation, personalized care, and the rise of chronic conditions were. Although scholars debate whether population aging is a key driver of HCE growth, their arguments suggest that population aging leads to some degree of HCE growth.

Regarding the impact of aging on the sustainability of medical insurance fund, most scholars believe that population aging poses certain challenges to the sustainable operation of medical insurance fund. Some of these scholars point out that population aging puts pressure on fund expenditure through increased HCE, which in turn affects the sustainability of medical insurance fund. The study of Colombier (18) illustrated that an aging population is a relevant determinant of HCE in Switzerland and will threaten the financial sustainability of the health system. Cubanski et al. (19) argued that future Medicare spending will grow faster than in recent years, partly due to increased services use as a result of increased number of insured people, and rising healthcare prices. Qiu et al. (20) stated that the degree of population aging in China will continue to intensify, the proportion of income of the medical insurance fund will gradually decrease and the aging population may threaten the balance of the medical insurance fund. Cristea et al. (21) identified that the older adults population’s increasing demand for medical services leads to the increasing *per capita* medical cost, which brings serious payment pressure to the social security system including medical insurance. Breyer and Lorenz (22) pointed out that in the process of population aging, there will be an increasing shift from active to retired people, which may make the social security system unsustainable.

In view of the fact that population aging can have some impact on fund sustainability, scholars have conducted research on relevant countermeasures to improve the sustainability of medical insurance fund. Scholars have mainly proposed various options from the fund income and expenditure sides, such as adjusting the maternity policy, financing models, implementing outpatient coordination reform, implementing delayed retirement policies. Liaropoulos and Goranitis (23) argued for the financing of comprehensive national health insurance through a progressive tax on income from all sources, spreading the cost of health care across all factors of production to ensure the sustainability of health system, especially in times of economic recession. Qian et al. (24) proposed to encourage appropriate fiscal decentralization to help maintain the sustainability of the social security fund. Zhang et al. (25) found that the implementation of China’s two-child policy helped to improve the process of population aging, delayed the onset of the urban residents’ basic medical insurance fund deficit and improved the sustainability of the basic medical insurance fund. Ren and Yang (26) suggested that China should implement outpatient mutual-aid guarantee mechanism as soon as possible to improve the solvency of the medical insurance fund for urban employees and establish an independent long-term care insurance system to ensure the stability and sustainability of the long-term care insurance fund and the medical insurance fund.

In summary, the above studies provide theoretical basis and empirical reference for this paper. So far, most scholars have analyzed the impact on the sustainability of the medical insurance fund from the changes and adjustments in one aspect of income or expenditure, and then propose policy options to promote the sustainable development of the fund. Few studies have examined the sustainability of China’s basic medical insurance fund for employees in terms of adjustments to two parameters (population age structure and growth rate of medical expenses). Furthermore, few studies have explored the sustainability of the medical insurance fund by taking a specific region as an example. Therefore, based on the reality of the accelerated aging process, this paper takes Shanghai basic medical insurance for employees as an example, and constructs an actuarial model with 2019 as the base year according to internal data from Shanghai Healthcare Security Bureau. Under different population projection scenarios and changes in the growth rate of *per capita* medical expenses due to non-demographic factors, the paper measures the income and expenditure and balance of pooling fund from 2021 to 2035, providing empirical support for the sustainable operation of China’s basic medical insurance fund for employees, with a view to promoting the sustainable development of the fund and reducing the burden on enterprises.

## 3. Materials and methods

### 3.1. Model

According to the policy, China’s basic medical insurance fund for employee is a combination of pooling fund and individual account. This means that the balance of the individual account is non-negative, the analysis in this paper is therefore aimed at pooling fund, which have a risk-sharing role.

#### 3.1.1. Pooling fund income model

The number of insured active employees multiplied by the contribution base multiplied by the contribution rate multiplied by the proportion of the fund income transferred to pooling fund equals to pooling fund income.^2^ The specific formula is as follows:


(1) $$\begin{array}{l} {\left(AI\right)}_{t}=\left(\overset{a_{t}^{m}-1}{\underset{x=22}{\sum }}N_{t,x}^{m}+\overset{a_{t}^{f,l}-1}{\underset{x=22}{\sum }}N_{t,x}^{f,l}+\overset{a_{t}^{f,w}-1}{\underset{x=22}{\sum }}N_{t,x}^{f,w}\right)\\ \;\;\;\;\;\;\;\;\;\;\;\;\;\;\;\cdot p_{t}\cdot {\bar{w}}_{2019}\cdot \overset{t}{\underset{s=2020}{\prod }}\left(1+k_{s}^{1}\right)\cdot R_{t}^{1}\cdot R_{t}^{2} \end{array}$$

(*AI*)*_t_* represents income of the basic medical insurance pooling fund for employees in year *t*. $N_{t,x}^{m}$ , $N_{t,x}^{f,l}$ 及 $N_{t,x}^{f,w}$ represent the number of employed urban males, female cadres and female workers in year t aged x respectively. $a_{t}^{m}$ , $a_{t}^{f,l}$ 及 $a_{t}^{f,w}$ refer to the legal retirement ages for males, female cadres and female workers in year t respectively. *p_t_* represents the insurance participation rate of active employees in year t . ${\bar{w}}_{2019}$ is the actual contribution base of active employees in 2019. $k_{s}^{1}$ represents the growth rate of the actual contribution base of active employees in year s . $R_{t}^{1}$ denotes the legal contribution rate of basic medical insurance for employees in year t . $R_{t}^{2}$ refers to the proportion of income of the basic medical insurance fund for employees transferred into pooling fund.

#### 3.1.2. Pooling fund expenditure model

The number of insured employees multiplied by the *per capita* expenditure of pooling fund equals the expenditure of pooling fund. The specific formula is as follows:


(2) $$\begin{array}{l} {\left(AC\right)}_{t}=\left(\overset{100}{\underset{x=22}{\sum }}N_{t,x}^{m}+\overset{100}{\underset{x=22}{\sum }}N_{t,x}^{f,l}+\overset{100}{\underset{x=22}{\sum }}N_{t,x}^{f,w}\right)\\ \;\;\;\;\;\;\;\;\;\;\;\;\;\;\;\;\cdot p_{t}\cdot {\bar{PAC}}_{2019}\cdot \overset{t}{\underset{s=2020}{\prod }}\left(1+k_{s}^{2}\right) \end{array}$$

(*AC*)*_t_* represents the expenditure of the basic medical insurance pooling fund for employees in year t . ${\bar{PAC}}_{2019}$ is the *per capita* pooling fund expenditure in 2019. $k_{s}^{2}$ is the growth rate of *per capita* pooling fund expenditure (or *per capita* medical expenses^3^) in year s .

#### 3.1.3. Cumulative balance model of pooling fund

The cumulative balance of the previous year is calculated at the bank’s three-month lump-sum deposit interest rate (*r_w_*), and the current balance is calculated at the bank’s current deposit interest rate (*r_t_*).^4^ The specific calculation formula for the cumulative balance of pooling fund is as follows:


(3) $$F_{t}=F_{t-1}\cdot \left(1+r_{w}\right)+C_{t}\cdot \left(1+r_{t}\right)$$

*F_t_* is the cumulative balance of the basic medical insurance pooling fund for employees at the end of year t . *C_t_* is the current balance of the basic medical insurance pooling fund for employees in the year t , i.e., pooling fund income minus pooling fund expenditure.

### 3.2. Parameter setting

#### 3.2.1. Number of insured employees and fertility rate

Using cohort component method,^5^ this paper forecasts the trends of resident population in Shanghai from 2021 to 2035,^6^ taking 2019 as the base year of the forecast. Then, according to different parameter settings, low, medium and high scenarios for total population projections are derived,^7^ as shown in Table 2. Among them, high population projection scenario will break the population control line of 25 million resident population.^8^

**Table 2:** Total resident population projections from 2021 to 2035 in Shanghai.

Year	Low scenario	Medium scenario	High scenario
2021	24.4882	24.5121	24.6209
2022	24.5674	24.6131	24.8272
2023	24.6278	24.7019	25.0553
2024	24.669	24.7776	25.3046
2025	24.6903	24.8497	25.5744
2026	24.6956	24.9171	25.8538
2027	24.6855	24.9803	26.1431
2028	24.6594	25.0385	26.442
2029	24.6174	25.0922	26.7507
2030	24.561	25.1429	27.0712
2031	24.4901	25.1794	27.3799
2032	24.4057	25.2033	27.6781
2033	24.3087	25.2151	27.9668
2034	24.1991	25.2153	28.2461
2035	24.0791	25.2059	28.5183

The underlying data used for forecasting comes from Shanghai National Economic and Social Development Statistical Bulletin, Shanghai Public Security Bureau Population Management Office. Unit: million.

**Figure 1:**
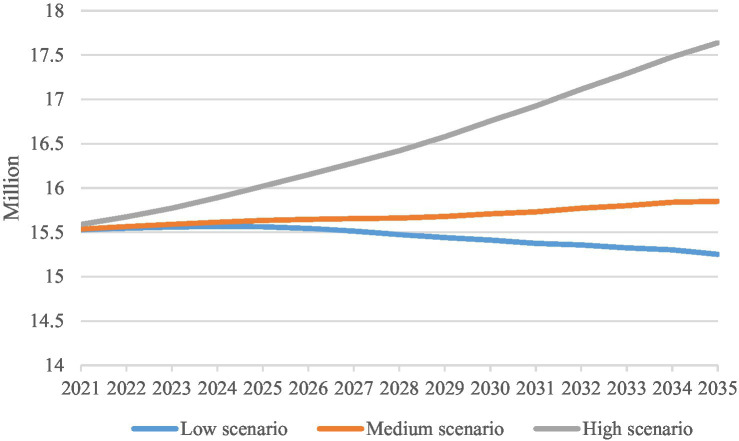
Forecast of the total number of participants of Shanghai basic medical insurance for employees from 2021 to 2035 (unit: million). The underlying data used for forecasting comes from 2019 Shanghai National Economic and Social Development Statistical Bulletin, Shanghai Public Security Bureau Population Management Office, China Statistical Yearbook 2020.

Based on the number of resident population by gender and five-year age group (19 years old and below as a group) in 2019, and the number of urban employees insured by gender and five-year age group (19 years old and below as a group) in 2019 as published in the National Bureau of Statistics of the People's Republic of China (27), the participation rate of Shanghai’s resident population by gender and five-year age group in 2019 can be calculated, and this participation rate will be used as a fixed extrapolation of the participation rate for the forecast period 2021–2035. Then, the participation rate is multiplied by the previously predicted population by gender and five-year age group in 2021–2035 to obtain the number of insured employees by gender and five-year age group and the total number of insured employees in 2021–2035. Consequently, the total number of participants of Shanghai basic medical insurance for employees from 2021 to 2035 under the low, medium and high scenarios can be projected, as shown in Figure 1.

As regards the total fertility rate(TFR), this paper sets the TFRs in urban and rural areas under the low scenario as 0.8 and 0.89 respectively; the TFRs in urban and rural areas under the medium scenario are 1.05 and 1.17 respectively; the TFRs in urban and rural areas under the high scenario are 1.3 and 1.44, respectively.

#### 3.2.2. Age

This paper sets the initial age of insurance participation as 20 years old,^9^ and the maximum survival age as 100 years old.^10^ Considering that Shanghai has not yet introduced a policy to delay the retirement age, the current policy will be adopted, with the retirement age set at 60 for men, 55 for female cadres and 50 for female workers.

#### 3.2.3. Actual contribution base

The actual contribution base in 2019 is 103906.76 yuan.^11^ It is assumed that growth rate of the actual contribution base for urban employees in Shanghai will be 5% in the future.^12^

#### 3.2.4. Legal contribution rate

The legal contribution rate of Shanghai basic medical insurance for employees was previously 14%, which was gradually reduced. The current legal contribution rate of Shanghai medical insurance is 11.5%, with the enterprise contribution rate at 9.5% and the individual contribution rate at 2%.

#### 3.2.5. Growth rate of *per capita* pooling fund expenditure

This paper refers to the “growth factor” method^13^ proposed by Mayhew (28) to predict the future growth rate of *per capita* pooling fund expenditure (or *per capita* medical expenses). On the premise of this assumption, the relationship between the growth factors and the changes in *per capita* pooling fund expenditure can be expressed as follows:


(4) $$H\left(t\right)=H\left(0\right)\times \exp^{t\left(rp+ru\right)}$$

**Figure 2:**
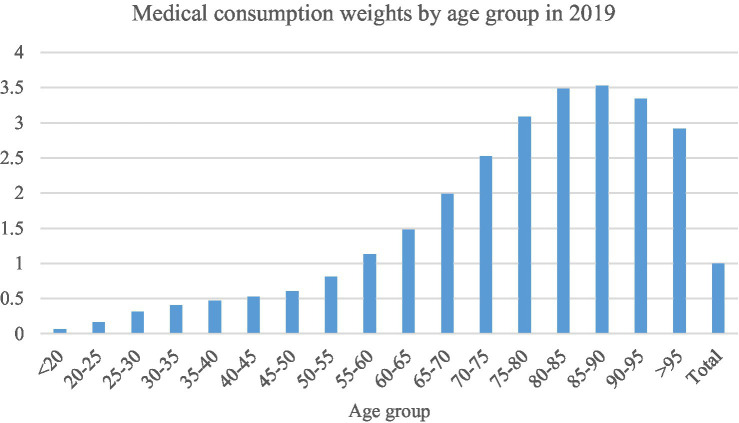
Medical consumption weights by age group in 2019. Data source: Shanghai Healthcare Security Bureau.

**Figure 3:**
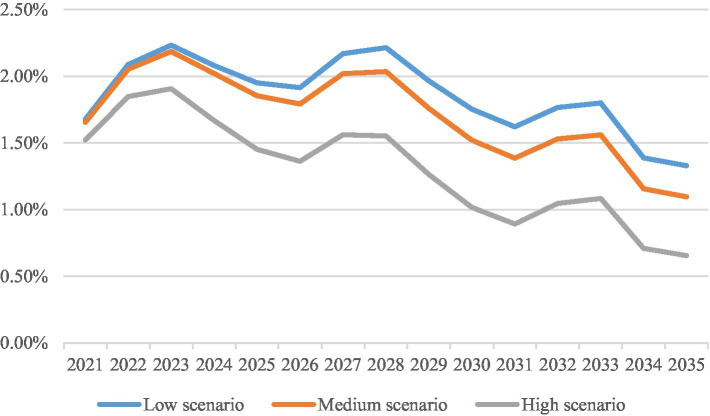
Growth rate of *per capita* medical expenses due to demographic factors from 2021 to 2035. Data source: Shanghai Healthcare Security Bureau.

*H*(0) is the *per capita* pooling fund expenditure in the base period, *H*(*t*) is the *per capita* pooling fund expenditure in time t , rp is the growth rate of *per capita* pooling fund expenditure due to demographic factors, ru is the growth rate of *per capita* pooling fund expenditure due to non-demographic factors. The sum of rp and ru is the overall growth rate of *per capita* pooling fund expenditure (or *per capita* medical expenses). According to the data of Shanghai Healthcare Security Bureau, the *per capita* pooling fund expenditure of Shanghai basic medical insurance for employees in 2019 was 2685.39 yuan.^14^ On this basis, the *per capita* pooling fund expenditure of each year can be obtained.^15^ Figure 2 reflects the medical consumption weights by age group in Shanghai in 2019,^16^ so that the growth rate of *per capita* medical expenses due to demographic factors for 2021–2035 under the low, medium and high population projection scenarios can be calculated,^17^ as shown in Figure 3. The growth rate of *per capita* pooling fund expenditure due to non-demographic factors has always been about 1% higher than the growth rate of the actual contribution base. However, given the advanced level of medical technology in Shanghai, there is still potential for growth in the growth rate of *per capita* pooling fund expenditure brought about by non-demographic factors. This paper assumes that the growth rate of *per capita* pooling fund expenditure due to non-demographic factors is 2% faster than the growth rate of the actual contribution base, 1% faster than the growth rate of the actual contribution base, as well as equal to the growth rate of the actual contribution base, so that the future growth rate of *per capita* pooling fund expenditure can be calculated.

#### 3.2.6. Bank interest rate

Based on data released by the People’s Bank of China, this paper sets the interest rate of current deposit as 0.35% and the interest rate of three-month lump-sum deposit as 1.35%.

## 4. Empirical results and analysis

Under three population projection scenarios (“low,” “medium” and “high”) and changes in the growth rate of *per capita* medical expenses due to non-demographic factors (“the growth rate of *per capita* medical expenses due to non-demographic factors is 2% faster than the growth rate of the actual contribution base,” “the growth rate of *per capita* medical expenses due to non-demographic factors is 1% faster than the growth rate of the actual contribution base,” and “the growth rate of *per capita* medical expenses due to non-demographic factors is equal to the growth rate of the actual contribution base”), this paper analyzes the operation of Shanghai basic medical insurance fund for employees from 2021 to 2035. As the data of Shanghai long-term care insurance is not available in this paper, research limitation resides in the fact that the impact of long-term care insurance on Shanghai basic medical insurance fund expenditure for employees^18^ has not been measured and studied. We will further investigate the data of Shanghai long-term care insurance to compensate for this research limitation in the future.

### 4.1. Forecast of pooling fund income and expenditure

#### 4.1.1. In low population projection scenario

Table 3 predicts the income and expenditure of Shanghai basic medical insurance pooling fund for employees in 2021–2035 under the changes of the growth rate of *per capita* medical expenses due to non-demographic factors in low population projection scenario. When the growth rate of *per capita* medical expenses due to non-demographic factors is 2% faster than the growth rate of the actual contribution base, the income and expenditure of pooling fund will continue to expand from 2021 to 2035, with income increasing from 79.046 billion yuan in 2021 to 130.413 billion yuan in 2035, with an average annual growth rate of 3.64%. Expenditure will increase from 49.19 billion yuan in 2021 to 158.923 billion yuan in 2035, with an average annual growth rate of 8.74%, far exceeding the growth rate of fund income. In 2031, the income of pooling fund will be unable to offset its expenditure.

**Table 3:** Forecast results of income and expenditure of Shanghai basic medical insurance pooling fund for employees from 2021 to 2035 (low scenario).

Year	When the growth rate of *per capita* medical expenses due to non-demographic factors is 2% faster than the growth rate of the actual contribution base		When the growth rate of *per capita* medical expenses due to non-demographic factors is 1% faster than the growth rate of the actual contribution base		When the growth rate of *per capita* medical expenses due to non-demographic factors is equal to the growth rate of the actual contribution base	
	Pooling fund income	Pooling fund expenditure	Pooling fund income	Pooling fund expenditure	Pooling fund income	Pooling fund expenditure
2021	79.046	49.19	79.046	48.289	79.046	47.395
2022	82.335	53.73	82.335	52.261	82.335	50.82
2023	85.173	58.738	85.173	56.61	85.173	54.54
2024	88.447	64.102	88.447	61.213	88.447	58.429
2025	91.764	69.825	91.764	66.066	91.764	62.477
2026	95.113	75.954	95.113	71.205	95.113	66.713
2027	98.708	82.762	98.708	76.877	98.708	71.362
2028	102.049	90.158	102.049	82.98	102.049	76.315
2029	105.615	98.027	105.615	89.395	105.615	81.453
2030	109.29	106.41	109.29	96.147	109.29	86.793
2031	113.084	115.307	113.084	103.227	113.084	92.318
2032	117.225	125.265	117.225	111.111	117.225	98.446
2033	121.389	135.994	121.389	119.519	121.389	104.913
2034	125.922	147.192	125.922	128.167	125.922	111.457
2035	130.413	158.923	130.413	137.104	130.413	118.118

Unit: billion yuan.

The change in the growth rate of *per capita* medical expenses due to non-demographic factors will not affect the income of pooling fund, but only the expenditure of pooling fund. No matter how the growth rate of *per capita* medical expenses due to non-demographic factors changes, the income of pooling fund from 2021 to 2035 is consistent. Under the circumstance that the growth rate of *per capita* medical expenses due to non-demographic factors is 1% faster than the growth rate of the actual contribution base, pooling fund expenditure will also increase year by year from 2021 to 2035, with an average annual growth rate of 7.74%, 4.10 percentage points higher than the average annual growth rate of fund income. By 2034, pooling fund will be unable to make ends meet. However, compared with the previous situation, expenditure will decrease by 1.83–13.73% from 2021–2035. When the growth rate of *per capita* medical expenses due to non-demographic factors is equal to the growth rate of the actual contribution base, pooling fund expenditure will expand from 47.395 billion yuan in 2021 to 118.18 billion yuan in 2035, with an average annual growth rate of 5.74%. Although the growth rate of expenditure is higher than the growth rate of income (3.10%), pooling fund will achieve balance of income and expenditure from 2021 to 2035. Compared with the first case, the expenditure of pooling fund will be further reduced by 3.65 to 25.68% from 2021–2035.

It can be seen that in low population projection scenario, when the growth rate of *per capita* medical expenses due to non-demographic factors is at the same level as the growth rate of the actual contribution base, the improvement effect of the income and expenditure of Shanghai basic medical insurance pooling fund for employees in 2021–2035 will be more obvious, which can realize the sustainable operation of the fund.

#### 4.1.2. In medium population projection scenario

As shown in Table 4, the income and expenditure of pooling fund will expand from 2021 to 2035 under the changes in the growth rate of *per capita* medical expenses due to non-demographic factors. When the growth rate of *per capita* medical expenses due to non-demographic factors is 2% faster than the growth rate of the actual contribution base, the income and expenditure of pooling fund will be 138.713 billion yuan and 161.633 billion yuan, respectively, in 2035, and the growth rate of expenditure (8.87%) is higher than the growth rate of income (4.09%). In 2032, income will not cover expenditure, and the gap between income and expenditure will gradually widen.

**Table 4:** Forecast results of income and expenditure of Shanghai basic medical insurance pooling fund for employees from 2021 to 2035 (medium scenario).

Year	When the growth rate of *per capita* medical expenses due to non-demographic factors is 2% faster than the growth rate of the actual contribution base		When the growth rate of *per capita* medical expenses due to non-demographic factors is 1% faster than the growth rate of the actual contribution base		When the growth rate of *per capita* medical expenses due to non-demographic factors is equal to the growth rate of the actual contribution base	
	Pooling fund income	Pooling fund expenditure	Pooling fund income	Pooling fund expenditure	Pooled fund income	Pooling fund expenditure
2021	79.111	49.204	79.111	48.302	79.111	47.408
2022	82.472	53.759	82.472	52.29	82.472	50.847
2023	85.416	58.791	85.416	56.661	85.416	54.588
2024	88.832	64.189	88.832	61.296	88.832	58.507
2025	92.385	69.97	92.385	66.202	92.385	62.604
2026	96.051	76.18	96.051	71.415	96.051	66.907
2027	100.058	83.098	100.058	77.185	100.058	71.644
2028	103.915	90.636	103.915	83.415	103.915	76.71
2029	108.114	98.689	108.114	89.991	108.114	81.99
2030	112.554	107.301	112.554	96.943	112.554	87.502
2031	117.19	116.466	117.19	104.252	117.19	93.223
2032	122.252	126.735	122.252	112.399	122.252	99.573
2033	127.421	137.822	127.421	121.106	127.421	106.29
2034	133.043	149.432	133.043	130.094	133.043	113.112
2035	138.713	161.633	138.713	139.414	138.713	120.084

Unit: billion yuan.

When the growth rate of *per capita* medical expenses due to non-demographic factors is 1% faster than the growth rate of the actual contribution base, the growth rate of the pooling fund expenditure from 2021 to 2035 will reach 7.87%, faster than the growth rate of income, and pooling fund will have an excess of expenditure over income in 2035. In contrast to the first case, expenditure will decrease by 1.83–13.75% from 2021–2035. Furthermore, when the growth rate of *per capita* medical expenses due to non-demographic is equal to the growth rate of the actual contribution base, expenditure will be 120.084 billion yuan in 2035, with an average annual growth rate of 6.86%, which also exceeds the growth rate of income. Nevertheless, pooling fund can achieve balance of income and expenditure in 2021–2035. Compared with the first situation, expenditure will decrease by 3.65–25.71% from 2021 to 2035.

As a consequence, under this scenario, the lower the growth rate of *per capita* medical expenses due to non-demographic factors, the better the sustainable operation effect of the fund. When the growth rate of *per capita* medical expenses due to non-demographic factors is the same as the growth rate of the actual contribution base, pooling fund can achieve medium and long term sustainability.

#### 4.1.3. In high population projection scenario

As can be seen from Table 5, the income and expenditure of pooling fund will continue to expand from 2021 to 2035 under high population projection scenario and changes in the growth rate of *per capita* medical expenses due to non-demographic factors. When the growth rate of *per capita* medical expenses due to non-demographic factors is 2% faster than the growth rate of the actual contribution base, income and expenditure will reach 163.36 billion yuan and 169.961 billion yuan, respectively, in 2035. The average annual growth rate of expenditure (9.25%) is far higher than the growth rate of income (5.28%), and until 2034 income will not be able to offset expenditure.

**Table 5:** Forecast results of income and expenditure of Shanghai basic medical insurance pooling fund for employees from 2021 to 2035 (high scenario).

Year	When the growth rate of *per capita* medical expenses due to non-demographic factors is 2% faster than the growth rate of the actual contribution base		When the growth rate of *per capita* medical expenses due to non-demographic factors is 1% faster than the growth rate of the actual contribution base		When the growth rate of *per capita* medical expenses due to non-demographic factors is equal to the growth rate of the actual contribution base	
	Pooling fund income	Pooling fund expenditure	Pooling fund income	Pooling fund expenditure	Pooling fund income	Pooling fund expenditure
2021	79.496	49.284	79.496	48.379	79.496	47.484
2022	83.291	53.934	83.291	52.458	83.291	51.009
2023	86.863	59.111	86.863	56.965	86.863	54.879
2024	91.131	64.713	91.131	61.79	91.131	58.974
2025	95.736	70.758	95.736	66.939	95.736	63.294
2026	100.628	77.292	100.628	72.446	100.628	67.862
2027	106.044	84.603	106.044	78.568	106.044	72.913
2028	111.51	92.615	111.51	85.216	111.51	78.348
2029	117.533	101.231	117.533	92.284	117.533	84.054
2030	124.03	110.51	124.03	99.81	124.03	90.059
2031	130.905	120.442	130.905	107.771	130.905	96.334
2032	138.396	131.593	138.396	116.66	138.396	103.305
2033	146.19	143.691	146.19	126.207	146.19	110.715
2034	154.643	156.451	154.643	136.138	154.643	118.308
2035	163.36	169.961	163.36	146.52	163.36	126.137

Unit: billion yuan.

Under the situation that the growth rate of *per capita* medical expenses due to non-demographic factors is 1% faster than the growth rate of the actual contribution base, income and expenditure can maintain balance between 2021 and 2035. In 2035, expenditure will be 146.52 billion yuan, with an average annual growth rate of 8.24%, which also exceeds the growth rate of income. In contrast to the first case, expenditure in 2021–2035 will decrease by 1.84–13.79%. When the growth rate of *per capita* medical expenses due to non-demographic factors is equal to the growth rate of the actual contribution base, expenditure in 2035 will be 126.137 billion yuan, with an average annual growth rate of 7.23%. Although the expenditure growth rate is 1.95 percentage points faster than that of income, pooling fund will continue to maintain balance of income and expenditure from 2021 to 2035, and optimize the sustainability of the fund. Compared with the first situation，expenditure will decrease significantly from 2021–2035, with a decrease of 3.65 to 25.78%.

In conclusion, based on the high population projection scenario, when the growth rate of *per capita* medical expenses due to non-demographic factors is 1% faster than the growth rate of the actual contribution base and is equal to the growth rate of the actual contribution base, pooling fund can achieve balance of income and expenditure in 2021–2035. The lower the growth rate of *per capita* medical expenses due to non-demographic factors, the more significant the improvement effect of the income and expenditure of pooling fund.

### 4.2. Medium and long term balance results of pooling fund

#### 4.2.1. In low population projection scenario

Table 6 reflects the medium and long term balance results of pooling fund from 2021 to 2035 corresponding to the changes in the growth rate of *per capita* medical expenses due to non-demographic factors in low population projection scenario. When the growth rate of *per capita* medical expenses due to non-demographic factors is 2% faster than the growth rate of the actual contribution base, the current balance of pooling fund will gradually decrease from 2021 to 2030, until the current deficit appears in 2031, and the deficit will increase each year. The cumulative balance will increase year by year from 2021, and reach the peak in 2031. Although the current deficit will occur in 2031, the cumulative balance will peak in the same year because the interest earned on the cumulative balance in 2030 will be greater than the size of the current deficit in 2031. After 2031, in order to make up the current deficit, the cumulative balance will progressively decrease.

**Table 6:** Medium and long term balance results of Shanghai basic medical insurance pooling fund for employees from 2021 to 2035 (low scenario).

Year	When the growth rate of *per capita* medical expenses due to non-demographic factors is 2% faster than the growth rate of the actual contribution base		When the growth rate of *per capita* medical expenses due to non-demographic factors is 1% faster than the growth rate of the actual contribution base		When the growth rate of *per capita* medical expenses due to non-demographic factors is equal to the growth rate of the actual contribution base	
	Current balance of pooling fund	Cumulative balance of pooling fund	Current balance of pooling fund	Cumulative balance of pooling fund	Current balance of pooling fund	Cumulative balance of pooling fund
2021	29.856	244.857	30.757	246.184	31.65	247.503
2022	28.605	276.867	30.073	279.686	31.514	282.469
2023	26.436	307.133	28.564	312.126	30.634	317.024
2024	24.346	335.71	27.234	343.669	30.018	351.427
2025	21.939	362.258	25.698	374.096	29.287	385.56
2026	19.159	386.374	23.907	403.138	28.399	419.264
2027	15.946	407.592	21.831	430.487	27.346	452.366
2028	11.892	425.028	19.069	455.435	25.734	484.297
2029	7.588	438.38	16.22	477.86	24.162	515.082
2030	2.88	447.189	13.143	497.5	22.498	544.612
2031	−2.223	450.995	9.857	514.108	20.767	572.803
2032	−8.041	449.015	6.113	527.183	18.778	599.38
2033	−14.605	440.421	1.871	536.178	16.476	624.006
2034	−21.27	425.022	−2.245	541.163	14.465	646.945
2035	−28.51	402.15	−6.691	541.754	12.295	668.017

Unit: billion yuan.

When the growth rate of *per capita* medical expenses due to non-demographic factors is 1% faster than the growth rate of the actual contribution base, the current balance will decrease in 2021–2033, and the current deficit will appear in 2034, with the deficit increasing year by year. In 2021–2035, the cumulative balance will increase gradually, reaching 541.754 billion yuan in 2035, with an average annual growth rate of 5.80%. Although the current deficit will occur between 2034 and 2035, the cumulative balance will be still increasing, because the bank interest obtained from the cumulative balance of the previous year will exceed the current deficit. Compared with the first situation, the occurrence of the current deficit will be delayed for 3 years (=2034–2031), and the cumulative balance in 2035 will increase by 34.71%. When the growth rate of *per capita* medical expenses due to non-demographic factors is equal to the growth rate of the actual contribution base, the current balance will decrease progressively to 12.295 billion yuan in 2035. The cumulative balance will increase from 2021 to 2035, reaching 668.017 billion yuan in 2035, with an average annual growth rate of 7.35%. Compared with the first situation, the current deficit will not occur in 2021–2035, and the cumulative balance in 2035 will increase by 66.11%.

Hence, in low population projection scenario, when the growth rate of *per capita* medical expenses due to non-demographic factors is the same as the growth rate of the actual contribution base, pooling fund will not have a current deficit from 2021–2035, and the cumulative balance will continue to increase, which will significantly improve the sustainable operation of the fund.

#### 4.2.2. In medium population projection scenario

As shown in Table 7, when the growth rate of *per capita* medical expenses due to non-demographic factors is 2% faster than the growth rate of the actual contribution base, the current balance will decrease year by year from 2021 to 2031. In 2032, there will be a current deficit in pooling fund, and the size of deficit will gradually increase to 22.920 billion yuan in 2035, 5.11 times the current deficit in 2032(=22.920/4.483). The cumulative balance is 244.925 billion yuan in 2021, expanding each year to peak at 464.552 billion yuan in 2032, 1.90 times the cumulative balance in 2021 (=464.552/244.925). Although the current deficit will occur in 2032, the cumulative balance will reach the peak in the same year, because the interest generated by the cumulative balance deposited in the bank in the previous year can make up the current deficit in 2032. Since then, in order to cover the current deficit, the cumulative balance will begin to decrease to 433.23 billion yuan in 2035.

**Table 7:** Medium and long term balance results of Shanghai basic medical insurance pooling fund for employees from 2021 to 2035 (medium scenario).

Year	When the growth rate of *per capita* medical expenses due to non-demographic factors is 2% faster than the growth rate of the actual contribution base		When the growth rate of *per capita* medical expenses due to non-demographic factors is 1% faster than the growth rate of the actual contribution base		When the growth rate of *per capita* medical expenses due to non-demographic factors is equal to the growth rate of the actual contribution base	
	Current balance of pooling fund	Cumulative balance of pooling fund	Current balance of pooling fund	Cumulative balance of pooling fund	Current balance of pooling fund	Cumulative balance of pooling fund
2021	29.907	244.925	30.809	246.253	31.702	247.572
2022	28.713	277.045	30.182	279.865	31.625	282.65
2023	26.625	307.502	28.755	312.499	30.827	317.401
2024	24.643	336.383	27.537	344.351	30.325	352.117
2025	22.415	363.417	26.183	375.274	29.78	386.755
2026	19.871	388.264	24.637	405.063	29.144	421.222
2027	16.96	410.525	22.872	433.484	28.413	455.422
2028	13.278	429.392	20.499	459.907	27.204	488.869
2029	9.425	444.647	18.123	484.302	26.124	521.685
2030	5.253	455.921	15.611	506.506	25.052	553.868
2031	0.724	462.803	12.938	526.327	23.967	585.396
2032	−4.483	464.552	9.853	543.32	22.679	616.056
2033	−10.402	460.385	6.314	556.991	21.131	645.578
2034	−16.389	450.154	2.949	567.469	19.931	674.294
2035	−22.92	433.23	−0.701	574.426	18.629	702.091

Unit: billion yuan.

Under the circumstance that the growth rate of *per capita* medical expenses due to non-demographic factors is 1% faster than the growth rate of the actual contribution base, the current balance will be 30.809 billion yuan in 2021, and will decrease each year thereafter to 2.949 billion yuan in 2034, and there will be a deficit in 2035. The cumulative balance will increase from 246.253 billion yuan in 2021 to 574.426 billion yuan in 2035, 2.33 times the cumulative balance in 2021 (=574.426/246.253), with an average annual growth rate of 6.24%. Compared with the first case (2% faster than the growth rate of the actual contribution base), pooling fund will not have a current deficit from 2021 to 2034, and the cumulative balance in 2035 will increase by 32.59%.

When the growth rate of *per capita* medical expenses due to non-demographic factors is equal to the growth rate of the actual contribution base, the current balance in 2021 will be 31.702 billion yuan, and then fall back to 18.629 billion yuan in 2035. The cumulative balance will increase from 247.572 billion yuan in 2021 to 702.091 billion yuan in 2035, 2.84 times the cumulative balance in 2021 (=702.091/247.572), with an average annual growth rate of 7.73%. Compared with the second case (1% faster than the growth rate of the actual contribution base), the cumulative balance in 2035 increased by 22.22%.

To sum up, under the medium scenario, compared with the first situation (2% faster than the growth rate of the actual contribution base), the latter two situations can further improve the sustainability of pooling fund. The cumulative balance will increase from 2021 to 2035, and the lower the growth rate of *per capita* medical expenses due to non-demographic factors, the better the medium and long-term balance effect of pooling fund.

#### 4.2.3. In high population projection scenario

It can be seen from Table 8 that when the growth rate of *per capita* medical expenses due to non-demographic factors is 2% faster than the growth rate of the actual contribution base, the current balance of pooling fund will gradually decrease from 2021 to 2033, and the current deficit will appear in 2034. The cumulative balance will continue to increase from 2021 to 2035, with an average annual growth rate of 5.78%. Why is there a current deficit in the poolig fund in 2035 and still a peak in the cumulative balance in the same year? The reason is that the current deficit in 2035 is less than the interest earned by the cumulative balance deposited in the bank.

**Table 8:** Medium and long term balance results of Shanghai basic medical insurance pooling fund for employees from 2021 to 2035 (high scenario).

Year	When the growth rate of *per capita* medical expenses due to non-demographic factors is 2% faster than the growth rate of the actual contribution base		When the growth rate of *per capita* medical expenses due to non-demographic factors is 1% faster than the growth rate of the actual contribution base		When the growth rate of *per capita* medical expenses due to non-demographic factors is equal to the growth rate of the actual contribution base	
	Current balance of pooling fund	Cumulative balance of pooling fund	Current balance of pooling fund	Cumulative balance of pooling fund	Current balance of pooling fund	Cumulative balance of pooling fund
2021	30.213	245.33	31.117	246.661	32.013	247.983
2022	29.357	278.101	30.833	280.931	32.281	283.725
2023	27.752	309.705	29.898	314.726	31.984	319.651
2024	26.417	340.395	29.34	348.417	32.156	356.236
2025	24.978	370.056	28.797	382.019	32.443	393.601
2026	23.336	398.47	28.182	415.457	32.766	431.795
2027	21.44	425.364	27.475	448.637	33.13	470.871
2028	18.894	450.067	26.293	481.079	33.162	510.505
2029	16.302	472.502	25.25	512.912	33.479	550.994
2030	13.52	492.449	24.221	544.141	33.971	592.522
2031	10.464	509.597	23.134	574.703	34.572	635.214
2032	6.803	523.304	21.737	604.274	35.092	679.003
2033	2.499	532.876	19.984	632.485	35.475	723.769
2034	−1.807	538.257	18.506	659.594	36.336	770.003
2035	−6.602	538.898	16.839	685.397	37.223	817.751

Unit: billion yuan.

When the growth rate of *per capita* medical expenses due to non-demographic factors decreases, the medium and long term balance results of pooling fund from 2021 to 2035 will be further optimized. Under the circumstance that the growth rate of *per capita* medical expenses due to non-demographic factors is 1% faster than the growth rate of the actual contribution base, the current balance will decrease progressively from 2021 to 2035. The cumulative balance will expand from 2021–2035, reaching 685.397 billion yuan in 2035, with an average annual growth rate of 7.57%. Compared with the first situation (2% faster than the growth rate of the actual contribution base), pooling fund will not have a current deficit in 2035, and the cumulative balance in 2035 will increase by 27.18%.

When the growth rate of *per capita* medical expenses due to non-demographic factors is equal to the growth rate of the actual contribution base, the current and cumulative balance of pooling fund from 2021 to 2035 are more optimistic, showing an increasing trend year by year. In 2035, the current and cumulative balance will reach 37.223 billion yuan and 817.751 billion yuan respectively, with an average annual growth rate of 1.08 and 8.90%, respectively.

Thus, under the high scenario, when the growth rate of *per capita* medical expenses due to non-demographic factors is equal to the growth rate of the actual payment base, the medium and long term balance results of pooling fund from 2021–2035 is the best, and the role of improving the sustainability of the fund is the most obvious.

## 5. Conclusions and policy recommendations

Based on the background of China’s population aging, China’s basic medical insurance fund expenditure for employees may increase significantly. How will the sustainability of China’s basic medical insurance fund for employees develop in the future? Taking Shanghai as an example, this paper establishes an actuarial model and finds that: first, under the changes in the growth rate of *per capita* medical expenses due to non-demographic factors and the low, medium and high population projection scenarios, Shanghai basic medical insurance fund for employees will achieve the goal of sustainable operation from 2021 to 2035, and the cumulative balance in 2035 will be 402.150–817.751 billion yuan. Second, when the growth rate of *per capita* medical expenses due to non-demographic factors is lower, the sustainable operation effect of basic medical insurance fund for employees is better, and the cumulative balance is larger. This demonstrates that Shanghai basic medical insurance fund for employees will be sustainable for the next 15 years, which can further reduce the pressure on enterprises to contribute, which will lay the foundation for improving the basic medical insurance benefits for employees. Accordingly, this paper makes the following recommendations to improve the basic medical insurance benefits for employees.

### 5.1. Individual account transfer to local additional fund

Reform and improve the outpatient coordination guarantee mechanism of basic medical insurance for employees. Improve the method for crediting individual account, incorporate the portion of individual contribution and enterprise contribution transferred to individual account into the local additional fund, in order to upgrade the outpatient guarantee mechanism and efficiency. According to the Shanghai Healthcare Security Bureau data, in 2016–2018, the loss rate of Shanghai basic medical insurance additional fund for employees was between 4.48 and 20.85%. If both the individual and enterprise contribution portions of the individual account are included in additional fund, the current balance rate is between 18.62 and 23.15%. In 2018, the settlement amount of outpatient and emergency department of Shanghai basic medical insurance for employees reached 40.038 billion yuan, of which the deductible amount of the outpatient and emergency was 558 million yuan, and the number of people entering the deductible section reached 1.0692 million. If the deductible amount for outpatient and emergency services is lowered by 200 yuan, an additional 214 million yuan will need to be spent by the medical insurance fund, and if it is lowered by 300 yuan, an additional 321 million yuan will need to be spent, accounting for 1.21 to 1.81% of the additional fund income for the year.

### 5.2. Reduce the deductible amount of basic medical insurance for employees

Based on the Shanghai Employees’ Basic Medical Insurance Measures promulgated by the Shanghai Municipal People’s Government, the outpatient deductible amount of Shanghai basic medical insurance for employees is 1,500 yuan for active employees, 700 yuan for retirees under 70 years old, and 300 yuan for retirees aged 70 and above. The part above the deductible amount is reimbursed by the additional fund in proportion. In order to further enhance the outpatient coordination guarantee function, it is proposed to further reduce the outpatient and emergency deductible amount of basic medical insurance for employees, and reduce the level of out-of-pocket medical expenses for participants in outpatient and emergency services.

### 5.3. Upgrade maternity insurance benefits

According to the Shanghai Healthcare Security Bureau data, in 2019, the annual maternity medical expenses in Shanghai were 515.0359 million yuan, 152,499 people enjoyed the reimbursement of maternity medical expenses, and 3377.31 yuan *per capita* enjoyed the reimbursement of maternity medical expenses; The annual maternity allowance expenditure was 6,773,823,500 yuan, 171,366 people enjoyed the maternity allowance, and the *per capita* maternity allowance was 39528.40 yuan. According to the actual situation of maternity medical expenses, if the *per capita* maternity medical expenses reimbursement is increased by 0.5 times, that is, the *per capita* maternity medical expenses reimbursement is 5065.96 yuan, then the annual expected maternity medical expenses will be 772.5539 million yuan. If the *per capita* maternity medical expense reimbursement is doubled, that is, the *per capita* maternity medical expense reimbursement is 6754.61 yuan, it is estimated that the annual maternity medical expense will be 1030.0718 million yuan.

## Data availability statement

Publicly available datasets were analyzed in this study. This data can be found at: http://www.stats.gov.cn/sj/ndsj/.

## Author contributions

W-ZW and YZ conceived and organized the study, and responsible for obtaining the data and building the actuarial model. Z-QY was responsible for conducting the study, analyzing the data, and writing the first draft. L-PC was responsible for reviewing the literature and developing the figures. J-QQ was responsible for revising the manuscript. All authors participated in the writing of the manuscript and approved the final manuscript.

## Funding

This study was supported by the National Social Science Fund of China (Grant Number: 21AZD070) and the Young Scientists Fund of the Fundamental Research Funds for the Central Universities (Grant Number: 2722023BY016).

## Conflict of interest

The authors declare that the research was conducted in the absence of any commercial or financial relationships that could be construed as a potential conflict of interest.

## Publisher’s note

All claims expressed in this article are solely those of the authors and do not necessarily represent those of their affiliated organizations, or those of the publisher, the editors and the reviewers. Any product that may be evaluated in this article, or claim that may be made by its manufacturer, is not guaranteed or endorsed by the publisher.

## Footnotes

^1^According to international practice, a moderately aging society is defined as one in which the proportion of people over 60 years old in the total population exceeds 20% or the proportion of people over 65 years old exceeds 14%.

^2^According to the current policy, retired employees do not have to contribute to medical insurance.

^3^As the reimbursement rate within the policy has not changed for many years, the growth rate of *per capita* pooling fund expenditure is equal to the growth rate of *per capita* medical expenses.

^4^According to the “Decision of the State Council on the Establishment of the Basic Medical Insurance System for Urban Employees.”

^5^Based on the existing data of population by gender and age at a certain point, the population by gender and age at a certain point in the future is estimated according to the survival probability of population, women’s fertility rate, net immigration rate, etc.

^6^The total resident population of Shanghai at year-end 2019 was 24.2814 million as published in the 2019 Shanghai National Economic and Social Development Statistical Bulletin multiplied by the age structure of the resident population by gender and one-year group in 2019 provided by Shanghai Public Security Bureau Population Management Office to obtain the number of resident population by gender and one-year group as the base data for the projection.

^7^The low scenario is low fertility and high mortality; the medium scenario is a compromise scenario, with fertility and mortality between the low and high scenarios; and the high scenario is high fertility and low mortality.

^8^The Shanghai Urban Master Plan (2017–2035) provides for a resident population of around 25 million people as the target for 2035.

^9^The Labor Law of the People’s Republic of China sets the legal age of employment at 16 years old, but given that it is common in China for university students to join the workforce at the age of 22 after graduation and for high school graduates to join the workforce at the age of 18. In this paper, it is assumed that the number of first-time insured employees is 50/50 between university students and high school students, and the age of first-time insured is set at 20.

^10^As successive census data grouped the population aged 100 and over into one age group (100 years and over) for enumeration, this paper assumes that the maximum surviving age of the population is 100 years.

^11^Data from Shanghai Healthcare Security Bureau, The income of Shanghai medical insurance fund for employees in 2019 = 126905.33 million yuan, the number of insured contributors is 10.620335 million, thus the *per capita* contribution is 11949.277 yuan = (12690.53/10.620335), the actual contribution base is 103906.76 yuan = 11949.277/ (11.5%).

^12^According to the Outline of the 14th Five-Year Plan and 2035 Vision for National Economic and Social Development of Shanghai, the average annual growth rate of Shanghai’s GDP is expected to be around 5%, hence this paper assumes that the future growth rate of the actual contribution base for urban employees in Shanghai will be 5%.

^13^The “growth factor” method features a decomposition of the growth rate of *per capita* pooling fund expenditure into several independent factors, including the degree of population aging, advances in medical technology, and changes in the disease spectrum. The factors are assumed to be independent of each other (28,29).

^14^In 2019, the expenditure of Shanghai basic medical insurance fooling fund for employees is 41340.87 million yuan and the total number of insured employess is 15.394742 million, therefore the *per capita* expenditure of pooling fund in 2019 is 2685.39 yuan(= 41340.87/15.394742).

^15^Per capita pooling fund expenditure in year t = per capita pooling fund expenditure in year t-1 multiplied by (1 + growth rate of per capita medical expenses in year t).

^16^This is fixed as the medical consumption weights by age group in the forecast period of 2021-2035, and the medical consumption weights by age group is equal to the *per capita* medical expenses by age group divided by the *per capita* medical expenses of the total number of people.

^17^The growth rate of *per capita* medical expenses due to demographic factors in year t = the weighted average medical consumption weight in year t divided by the weighted average medical consumption weight in year t-1 minus 1. Weighted average medical consumption weight = the number of people in each age group multiplied by medical consumption weight in each age group, all summed up, and then divided by the total number of people.

^18^Shanghai began a pilot long-term care insurance scheme in 2016, which is mainly funded by basic medical insurance for employees and public finance. With Shanghai’s population set to age rapidly over the next 15 years, expenditure related to long-term care insurance will grow rapidly. In the absence of new funding sources, this will put new pressure on the expenditure of Shanghai basic medical insurance fund for employees.
